# Subjective, intersubjective, and objective social statuses: How do people imagine social inequality?

**DOI:** 10.1016/j.ssmph.2025.101878

**Published:** 2025-11-08

**Authors:** Naoki Sudo

**Affiliations:** Graduate School of Social Sciences, Hitotsubashi University, 2-1 Naka, Kunitachi-shi, Tokyo, 186-8601, Japan

**Keywords:** Subjective social status, Intersubjective social status, Objective social status, Social inequality of opportunities, Social inequality of outcomes

## Abstract

Previous studies have shown that although subjective social status is related to objective social status, these statuses do not necessarily coincide with each other. Consequently, the relationship between subjective social status and social inequality had not been sufficiently explored. Accordingly, this study explored how and why they are related to each other by introducing the new concept of intersubjective social status. To measure subjective and intersubjective social statuses, I analyzed data from the Stratification and Social Psychology Survey in 2022 (SSP2022) and an online survey experiment by using ordered and multi-level ordered logistic regression models. The analysis revealed that although subjective and intersubjective social statuses share basic features, they are not the same concept. Subjective social status emphasizes the social inequality of opportunities, whereas intersubjective social status emphasizes the social inequality of outcomes. Additionally, the results revealed that the association between subjective and intersubjective (or objective) social statuses might vary depending on social status groups.

## Highlights

• Intersubjective social status is defined as social status that is subjectively rated by others.
• Although subjective and intersubjective social statuses share basic features, they are not the same concept.
• Subjective social status emphasizes the social inequality of opportunities.
• Intersubjective social status emphasizes the social inequality of outcomes.
• The association between subjective and intersubjective social statuses varies depending on social status groups.

## 1 Introduction

Previous studies have stated that subjective social status is more significantly associated with individual well-being than objective social status (Adler et al., 2000). Additionally, previous studies have revealed that subjective social status is significantly associated with social inequality, and widening social inequality tends to hurt individual subjective social status and subjective well-being (Andersen & Curtis, 2012; Chen & Williams, 2018; Curtis, 2013). Considering a weak link between subjective and objective social status (Evans & Kelley, 2004; Oesch & Vigna, 2023), the association of subjective social status with social inequalities is a critical issue for social researchers. Nevertheless, why subjective social status does not coincide with objective social status, and why it is related to social inequality, has not been sufficiently examined in previous studies.

This study introduces the concept of intersubjective social status to clarify differences between subjective and objective social statuses with respect to their associations with social inequality. The comparison of subjective and intersubjective social status will provide clarity about the types of social inequality that are more related to subjective social status. As intersubjective social status (and objective social status) can be decided based only on information of the current statuses of the individuals (for instance, employment status), it will especially focus on inequality of outcomes. Subsequently, subjective social status can be determined based on information about the past and current statuses of the individuals simultaneously (for instance, educational career and employment status), it will focus not only on social inequality of outcomes, but also on social inequality of opportunities.

## 2 Literature review

Previous studies have pointed out that subjective social status has more positively affected subjective well-being and health than objective social status (Adler et al., 2000; Sakurai et al., 2010; Singh-manoux et al., 2003; Singh-Manoux et al., 2005; Varghese et al., 2022). This suggests that subjective social status can vividly reflect social contexts perceived by individuals as compared to objective social status, which is measured based on limited social indicators, and can become a more powerful predictor of subjective well-being and health. Additionally, previous studies (Andersen & Curtis, 2012; Curtis, 2016; Haddon, 2015; Kirsten et al., 2023; Lindemann & Saar, 2014; Nam, 2016; Nolan & Weisstanner, 2022; Oddsson, 2018; Schneider, 2019; Speer, 2016; Vigna, 2023; Zhou, 2021) have clarified that the association between subjective and objective social statuses may vary depending on social inequality. Consequently, subjective social status is also related to political preferences through perceived social inequality (Melli & Azzollini, 2025).

Nevertheless, how and why subjective social status is associated with social inequalities has not been sufficiently clarified, because subjective social status has a highly complex constituent. For instance, it is well known that an individuals' subjective social status can be related to the socioeconomic statuses not only of themselves, but also of their family members, such as partners or parents (Bavetta et al., 2019; Brunori, 2017; Davis & Robinson, 1988; Hodge & Trieman, 1968; Knudsen, 1988; Oesch & Vigna, 2023; Pérez-Ahumada, 2014; Ritter & Hargens, 1975). Moreover, compared with objective social status, the distribution of subjective social status tends to be concentrated at the middle points (Evans & Kelley, 2004; Schmalor & Heine, 2022; Sosnaud et al., 2013). Despite the middle-class tendency, subjective social status is associated with social inequalities in society (Andersson, 2015; Chen & Williams, 2018; Curtis, 2013). Similarly, the association between subjective social status and objective social status is not stable but varies with social change. For example, the fact that the association between subjective social status and objective social status was strengthened with the changes in Japanese society over the last four decades is well known among Japanese sociologists (Hommerich & Kikkawa, 2019; Kikkawa, 2000; Kikkawa & Fujihara, 2012; Sudo, 2019). These facts reveal complex constituents of subjective social status.

Previous studies have theoretically explained the complex constituents of subjective social status based on the reference group theory or similar theories (Aevar Oddsson, 2010; Evans et al., 1992; Fararo & Kosaka, 1992, 2003; Merton, 1957; Oh et al., 2022). However, although this type of explanation could discuss how and why subjective social status does not align with objective social status, it does not clarify how or why subjective social status is more strongly related to social inequalities and subjective well-being than objective social status. This study addresses this issue by introducing a new concept of intersubjective social status.

## 3 Theory and hypotheses

**Fig. 1:**
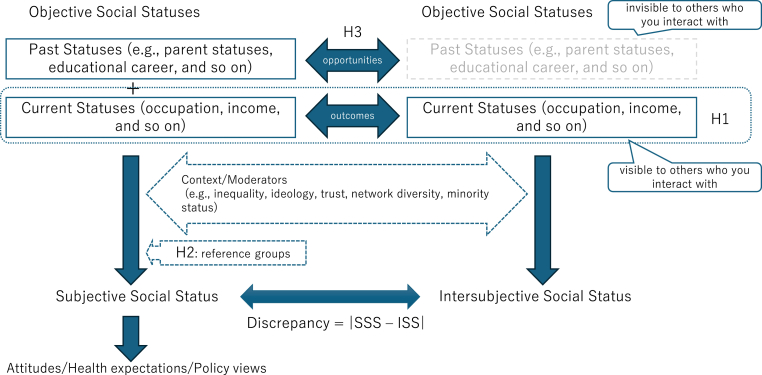
Conceptual diagram.

To explain complex associations among subjective social status, objective social status, and social inequality, I propose a theory based on the concept of intersubjective social status (see Table 1 and Fig. 1). Subjective social status can be considered as self-rated social status, whereas objective social status can be considered as social status measured by objective socioeconomic status (e.g., income, employment, job, and education). By contrast, intersubjective social status can be defined as individuals’ social status as rated by others. Thus, intersubjective social status is neither subjective nor objective social status.

**Table 1:** Subjective social status and intersubjective social status.

	Subjective social status	Intersubjective Social Status
Who rates?	Self	Other
Past Statuses	visible (utilized)	invisible (not utilized)
Current Statuses	visible (utilized)	visible (utilized)
Inequality of Opportunities	captured	not captured
Inequality of Outcomes	captured	captured

It is assumed that subjective social status is exposed to two types of bias. One of these can be derived from differences in reference groups (Kirsten et al., 2023). For instance, individuals objectively categorized into high-status groups may lower their subjective social status because high status is not high among them. The other bias can be derived from the shortage of information on objective social status. Objective social status judged by an individual based on limited information may not coincide with correct objective social status. The former type of bias is generated between subjective and intersubjective social statuses because it can be interpreted as a kind of self-centered bias. Conversely, the latter type of bias is generated between subjective and objective social statuses because it can be interpreted as a type of measurement error.

If the type of self-centered bias is eliminated among individuals, subjective and intersubjective social statuses will coincide more. Conversely, if the type of measurement error is eliminated, subjective and objective social statuses will coincide. However, as these types of biases are observed in subjective, intersubjective, and objective social statuses, discrepancies between subjective and objective social statuses could appear, even though subjective and objective social statuses are known to be associated with each other (Jackman, 1979; Jackman & Jackman, 1973; Vanneman & Pampel, 1977).

Under these assumptions, the following hypotheses are derived. First, as subjective social status is constructed based on intersubjective social status (social images held by each individual), the subjective social status for each individual will be the same as the intersubjective social status of individuals sharing socioeconomic statuses with that individual. Specifically, it is predicted that the pattern of association between the subjective social status of the individual and the objective social status of the individual is the same as the pattern of association between the individual's intersubjective status and the individual's objective social status. This prediction can be formulated as follows:

Hypothesis 1 The subjective social status of individuals (social status rated by themselves) shares basic features with their intersubjective social status (social status rated by others except the individual).

Second, irrespective of Hypothesis 1, individuals' subjective social status will not perfectly coincide with intersubjective social status. Individuals judge their subjective social status by referring to their reference groups (Huang, 2023, Wei, 2022). Therefore, individuals’ subjective social status can differ depending on the distributions of objective social status in their reference groups, even when individuals belonging to different groups share the same objective social status. For instance, socially advantaged individuals are more likely to report lower subjective social status than the subjective social status intersubjectively rated by members of a socially disadvantaged group. This prediction can be formulated as follows:

Hypothesis 2 Socially advantaged individuals are more likely to lower their subjective social status, and socially disadvantaged individuals are more likely to elevate their subjective social status. Owing to this tendency, the number of individuals who attribute themselves to the middle status group will increase (the middle-status tendency).

Third, irrespective of Hypothesis 1, subjective social status focuses not only on social inequality measured by objective social status (e.g., the social inequality of outcomes) but also on the dimensions of potential social inequality (e.g., the social inequality of opportunities). Hence, the discrepancy between subjective and intersubjective social statuses will be wider. Specifically, individuals simultaneously emphasize information for their current social status (e.g., employment status) and past social status (e.g., educational career) when deciding the status of themselves. However, these individuals consider only information about the other person's current social status when assessing the other person's status. This prediction can be formulated as follows:

Hypothesis 3 Individuals' subjective social status is based more on the social inequality of opportunities, whereas their intersubjective social status is based more on the social inequality of outcomes.

## 4 Data and methods

### 4.1 Data

To examine hypotheses presented in this study, I analyzed two datasets: the Stratification and Social Psychology Survey in 2022 (SSP2022) and data from an online experimental survey I conducted. SSP2022 is a national representative survey, which is one of the serial surveys conducted by the SSP project team in Japan. SSP2022 was conducted from January to March 2022, and the sample of SSP2022 was Japanese individuals selected from poll books administrated by each municipality based on a stratified multistage random sampling method. The sample size was 5995; however, the number of effective responses was 2395 (response rate: 39.95 %). The age ranged from 25 to 65 years.

I collected online experimental data in April 2025 to measure Japanese residents’ intersubjective social status. The experimental data sample was selected from online monitors registered in a research agency in Japan, Rakuten Insight (https://member.insight.rakuten.co.jp/) and the size of the sample was 804. The experimental data were not nationally representative of social survey data; therefore, the trends of (subjective) social status in Japan could not be investigated based on the experimental data. However, as the experimental data were implemented using the random assignment method, they could show the latent structure of (intersubjective) social status.

In this study, I examined the association between the subjective social status, demographic characteristics, and socioeconomic status of Japanese individuals in their 30s based on the SSP2022 data. Additionally, I examined the association between subjective social status, demographic characteristics, and socioeconomic status of individuals with fictional profiles based on experimental data. Thereafter, I compared the social structure of subjective social status, which has been clarified by SSP2022 data, and the social structures of intersubjective social status, which has been clarified by the experimental data, and explored the common or different characteristics between them. The combined survey-experimental design is a significant characteristic of this study. By combining the two data sets, I could specify common features of subjective social status and intersubjective social status, and effectively bring to light differences between them.

### 4.2 Variables

*Dependent variable*. The dependent variable in this study was subjective (or intersubjective) social status. First, the respondents of SSP2022 were requested to choose one of five alternatives (5. Upper, 4. Upper-Middle, 3. Lower-Middle, 2. Upper-Lower, or 1. Lower-Lower) according to their social status. These responses were defined as reflecting subjective social status. Next, the respondents of the survey experiment were presented with four profiles, including demographic characteristics (name, age, and marital status) and social status (educational level, job content, and employment status), and were asked to predict their subjective social status (5. Upper, 4. Upper-Middle, 3. Lower-Middle, 2. Upper-Lower, 1. Lower-Lower). These responses were defined as reflecting intersubjective social status. The subjective (or intersubjective) social status measured in this study was treated as a 5-point Likert scale variable.

*Independent variables*. Gender, marital status, education level, and employment status were treated as independent variables. For gender, the respondents of SSP2022 were categorized into three categories (men, women, and non-binary), and the individuals depicted in the fictional profiles used in the survey experiment were categorized into two categories (men and women). For marital status, the respondents of SSP2022 were categorized into three categories: unmarried, married, and divorce/bereaved, while the individuals depicted in the fictional profiles were categorized into two categories: unmarried and married. Regarding education level, the respondents of SSP2022 and the individuals depicted in the fictional profiles were divided into two categories: high school graduates and college graduates. Finally, for employment status, the respondents of SSP2022 were categorized into four categories (regular employment, non-regular employment, self-employment, and no job), and the individuals depicted in the fictional profiles were categorized into two categories: regular employment and non-regular employment. Regular employment means being a full-time worker and non-regular employment being a part-time worker or a temporary worker.

*Control variables*. When I implemented the online survey experiment, the information on the respondents’ demographic characteristics (gender) and social status (education level and employment status) were also collected. In this study, information on gender, education level, and employment status of the respondents in the experimental survey were treated as control variables. Regarding gender, the respondents in the survey experiment were divided into three categories (men, women, and non-binary). Regarding education level, the respondents of the survey experiment were categorized into two groups: high school graduates and college graduates. For employment status, the respondents in the survey experiment were divided into four categories (regular employment, non-regular employment, self-employment, and unemployment).

### 4.3 Analytical strategy

To confirm the structure of the subjective social status of Japanese individuals in their 30s based on SSP2022, I used the ordered logistic regression model to predict subjective social status. The regression model predicting the subjective social status of Japanese individuals in their 30s was formulated as follows:

$$\mathrm{Pr}\left(SSS_{j}=i\right)=\mathrm{Pr}\left(\kappa_{i-1}<\beta_{1}Gender+\beta_{2}Marital+\beta_{3}Education+\beta_{4}Employment+\varepsilon_{j}<\kappa_{i}\right)\text{,}$$

where SSS means subjective social status; *i* means the category of subjective social status; *j* means the respondent's ID number; κ means the cutoff point; *Gender* means the dummy variable of gender; *Marital* means the dummy variable of marital status; *Education* means the dummy variable of education level; *Employment* means the dummy variable of employment status; and ε means the error term.

Conversely, to confirm the structure of intersubjective social status of Japanese individuals based on the online survey experiment data, I used the multilevel ordered logistic regression model predicting for intersubjective social status. The regression model predicting the intersubjective social status of Japanese individuals can be formulated as follows:

$$\mathrm{Pr}\left(ISS_{jk}=i\right)=\mathrm{Pr}\left(\kappa_{i-1}<\beta X_{jk}+\gamma Z_{j}+\delta X_{jk}\cdot Z_{j}+\mu_{j}+\varepsilon_{jk}\leq \kappa_{i}\right)\text{,}$$

where *ISS* means intersubjective social status; *i* means the category of intersubjective social status; *j* means respondent's ID number; *k* means the presented profile; and κ means the cutoff point. Moreover, β means the coefficient vector of characteristics written in the profile; γ means the coefficient vector of characteristics for the respondent; and δ means the coefficient vector of interaction terms between characteristics in the profile and characteristics of the respondent. *X* is the vector of independent variables at the profile level and *Z* is the vector of independent variables at the respondent level. Finally, μ means the error term at the respondent level and ε means the error term at the profile level. If beta coefficients change significantly by adding variables for the respondent's characteristics and considering interaction with characteristics written in each vignette, it indicates that the cognitive process recognizes inter subjective social status is not universal but rather dependent on social context.

If Hypothesis 1 has empirical validity, the coefficient vector of the independent variables in the regression model predicting subjective social status and the coefficient vector of the independent variables at the profile level in the regression model predicting intersubjective social status will show similar tendencies. Similarly, if Hypothesis 2 has empirical validity, the coefficient vector of the independent variables at the respondent level in the regression model predicting intersubjective social status will be statistically significant. Finally, if Hypothesis 3 has empirical validity, systematic differences in the latent structure between subjective and intersubjective social statuses will be observed.

To estimate the coefficients of the independent variables and variances of the error term, I used the R language and one of its packages, *ordinal* (Christensen, 2023; R Core Team, 2018).

### 4.4 Descriptive statistics

Table 2 presents the descriptive statistics of variables in SSP2022. The table also shows the arithmetic mean, standard deviation, and median of the quantitative variables (subjective social status) and the percentages of dummy variables. Because the age of the persons in the files used in this study was fixed at 35, the respondents in SSP2022 were also restricted to those in their 30s to compare with intersubjective status and subjective status. Consequently, the number of cases was 504. The arithmetic mean of subjective social status was 2.95, the standard deviation was 0.93, and the median was 3.0. The marriage rate among all respondents was 62 %. Conversely, the percentage of college graduates was 0.58. Compared with other cohorts, these (the low rate of marriage and the high rate of college graduates) were prominent characteristics of the cases used in this study, who were in their 30s. These characteristics should be considered carefully when examining the subjective social status of individuals in their 30s.

**Table 2:** Descriptive statistics of variables in SSP2022.

	Mean	SD	Median	Min	Max
Subjective Social Status	2.95	0.93	3	1	5
	N	%			
Men	237	47.0			
Women	268	53.0			
Non-Binary	2	0.0			
Married	314	62.1			
Unmarried	172	33.7			
Divorce/Bereaved	21	4.2			
College Graduate	295	58.3			
Regular Employment	312	61.7			
Non-regular Employment	88	17.4			
Self Employed	37	7.1			
No Job	53	10.5			
No Information for Employment Status	17	3.4			

N = 506.

Table 3 presents the descriptive statistics of variables in the online survey experiment. It also shows the arithmetic mean, standard deviation, and median of quantitative variable (intersubjective social status) and the percentages of dummy variables at the profile level in all responses as well as the percentages of dummy variables at the respondent level. The number of responses was 3,205, and the number of respondents was 804. The arithmetic mean of intersubjective social status was 3.00, the standard deviation was 0.84, and the median was 3.0. The arithmetic mean and standard deviation of intersubjective social status measured in the online survey experiment were values similar to those of subjective social status reported in SSP2022. The percentage of women respondents was 32 %, which means that women respondents were clearly underrepresented in the online experiment data. Additionally, the percentage of unemployed respondents in the online survey experiment was 25 %, which is lower than the rate of unemployed respondents in SSP2022. Therefore, these characteristics should be carefully considered when interpreting the analytical results based on the online survey experiment.

**Table 3:** Descriptive statistics of variables in the survey experiment data.

	Mean	SD	Median	Min	Max
Profile (N = 3205)					
Intersubjective Social Status	3.00	0.84	3	1	5

	N	%			

Women	1601	50.0			
Married	1402	43.7			
College Graduate	1802	56.2			
Regular Employment	1799	56.1			

Respondents (N = 804)	N	%			

Women	256	31.8			
College Graduate	404	50.2			
No-information of Educational Status	9	1.1			
Regular Employment	347	43.1			
Non-regular Employment	170	21.1			
Self-employed	76	9.4			
No Job	205	25.5			
No Information of Employment Status	7	0.9			

Note: 3205 represents the number of responses, while 804 refers to the number of respondents.

## 5 Analytic results

### 5.1 Differences in distribution of subjective social status between experimental data and SSP2022

**Fig. 2:**
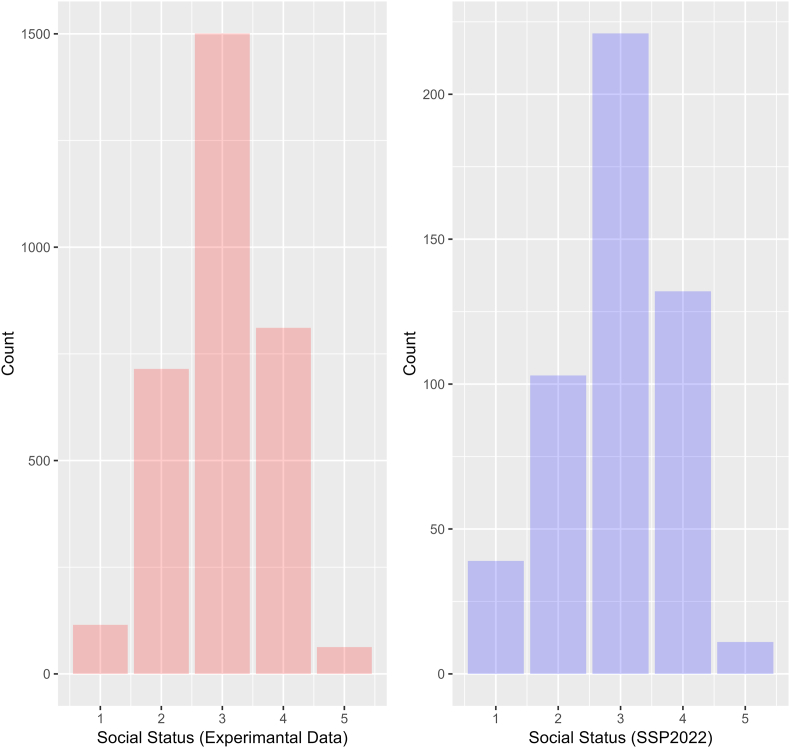
Distributions of social status (Left: Experimental data. Right: SSP2022 data).

First, I compared the distribution of subjective social status in SSP2022 and intersubjective social status in the online experiment. Fig. 2 shows the distribution of subjective and intersubjective social statuses. Interestingly, both exhibit similar characteristics. Specifically, the arithmetic means of subjective social status are not statistically different between the experimental data and SSP2022 (t = 1.1624, df = 641.4, p-value = 0.2455). Although the variances in subjective social status are statistically different (F (3204, 505) = 0.81359, p = 0.002), the shapes of distribution are similar. Given that the cognitive processes behind subjective and intersubjective social status differ, the two distributions cannot be compared directly. Nevertheless, the intersubjective social status measured in the online survey experiment may have been constituted like the subjective social status reported in social surveys.

To examine differences in subjective and intersubjective social statuses, I analyzed the association with subjective social status (or intersubjective social status) and independent variables by using a *t*-test. Table 4 presents the results. Regarding demographic variables (gender and marital status), gender (man/woman) has no statistically significant association with subjective social status. Similarly, gender was not significantly associated with intersubjective social status. Additionally, marital status has a statistically significant association with subjective and intersubjective social statuses. Married individuals are more likely to attribute themselves to a higher social status and are more likely to be considered individuals with a higher social status.

**Table 4:** Results of T -tests.

Variable	N	Mean	SD	Min	Max	p-value	Sig.
Experimental Data (Dependent Variable: Intersubjective Social Status)							
Men	1604	2.98	0.838	1	5	0.151	
Women	1601	3.02	0.833	1	5		
Unmarried	1803	2.87	0.823	1	5	0.000	∗∗∗
Married	1402	3.16	0.824	1	5		
High School	1403	2.84	0.789	1	5	0.000	∗∗∗
College Graduate	1802	3.12	0.852	1	5		
Non-regular	1406	2.69	0.788	1	5	0.000	∗∗∗
Regular	1799	3.24	0.792	1	5		

SSP2022 Data (Dependent Variable: Subjective Social Status)							
Men et al.	238	3.00	1.010	1	5	0.228	
Women	268	2.90	0.840	1	5		
Unmarried et al.	192	2.69	1.010	1	5	0.000	∗∗∗
Married	314	3.11	0.834	1	5		
High School	211	2.63	0.940	1	5	0.000	∗∗∗
College Graduate	295	3.18	0.847	1	5		
Non-regular et al.	194	2.79	0.921	1	5	0.003	∗∗
Regular	312	3.04	0.919	1	5		

+ p < 0.1, ∗p < 0.05, ∗∗p < 0.01, ∗∗∗p < 0.001.

Table 4 reveals that among the objective social status—education level and employment status—the former had a statistically significant association with subjective and intersubjective social statuses, and individuals with more than college degrees are more likely to attribute themselves to a higher social status and are more likely to be considered as members of a higher social status group simultaneously. Similarly, employment status showed a statistically significant association with subjective and intersubjective social statuses. In other words, regularly employed individuals are more likely to attribute themselves to a higher social status and to be considered as the members of a higher social status group. Taken together, the pattern of association between subjective social status and independent variables (demographic characteristics and objective social status) is similar to the pattern of association between intersubjective social status and independent variables.

### 5.2 Predicting subjective social status based on social survey data

I analyzed data from SSP2022 by using an ordered logistic regression model that predicts subjective social status to confirm the structure of subjective social status. Table 5 presents the analytical results of the ordered logistic regression model. In this model, only cases in their 30s were analyzed, and 506 cases were used in the analysis. As previously mentioned, the dependent variable in the ordered logistic regression model was subjective social status, which was measured using 5-point Likert scale. The independent variables in the ordered logistic regression model were dummy variables of gender, marital status, education level, and employment status.

**Table 5:** Results of ordered logistic regression model predicting subjective social status.

	Model 1
Women	−0.130
	(0.183)
Nonbinary	1.077
	(1.253)
Married	0.990∗∗∗
	(0.186)
Divorced/Bereaved	1.219∗∗
	(0.430)
College Graduates	1.053∗∗∗
	(0.178)
Regular Employment	0.735∗∗
	(0.241)
Self-employed	0.635+
	(0.365)
Unemployed	0.516
	(0.322)
No Information on Employment	1.181∗
	(0.491)
1|2	−0.942∗∗
	(0.296)
2|3	0.771∗∗
	(0.286)
3|4	2.891∗∗∗
	(0.313)
4|5	5.885∗∗∗
	(0.433)
Num. Obs.	506
AIC	1273.7
BIC	1328.7
RMSE	2.90

+ p < 0.1, ∗p < 0.05, ∗∗p < 0.01, ∗∗∗p < 0.001.

The analytical results of the ordered logistic regression model revealed that the variables of gender and employment status, except regular employment, were not significantly associated with subjective social status. However, the analytical results clarified that marital status, education level, and regular employment (vs. non-regular employment) were significantly associated with subjective social status. Additionally, the analytical results showed that all the cutoff points were statistically significant. In summary, the subjective social status of Japanese individuals in their 30s is ordered hierarchically based on objective social status (marital status, education level, and employment status).

Notably, for the subjective social status of Japanese individuals in their 30s, marital status seems to be included in objective social status. Unmarried individuals are less likely to attribute themselves to higher social-status groups. Moreover, I had to focus on differences in subjective social status between employment-status groups. Although significant differences in the association with subjective social status between individuals who have non-regular employment [reference], self-employment, and no job were not observed, only regular employment [vs. non-regular employment] was significantly and positively associated with subjective social status. This suggests that regular employment has significant implications for Japanese society. Lastly, Table 5 clarifies that even though the enrollment percentage in college is already over 50 % in Japanese society, a college degree still has significant implications for the subjective social status of Japanese individuals in their 30s, and the coefficient of college graduates is not significantly different from that of regular employment ($\chi^{2}$ = 1.036, df = 1, p = 0.31). To check robustness, I implemented the model using the full SSP 2022 sample. I verified that the main results remained unchanged and that the coefficient for college graduates is significantly larger than that for regular employment (χ^2^ = 12.59, df = 1, p < 0.001). This result supports Hypothesis 3 more strongly (see Table S1 in Supplemental Materials in detail).

### 5.3 Predicting intersubjective social status based on experimental data

To confirm the structure of intersubjective social status, I analyzed data from the online survey experiment by using a multi-level ordered logistic regression model predicting intersubjective social status. Table 6 presents the analytical results of the multilevel-ordered logistic regression model. The number of observations (profiles used for the prediction of intersubjective social status) was 3,205, and the number of groups (the respondents of the online survey experiment) was 804. Model 1 refers to a pooled ordered logistic regression model that predicts intersubjective social status based only on information written in the profile. Model 2 is a multilevel ordered logistic regression model that predicts intersubjective social status based only on information written in the profile. Model 3 refers to a multilevel ordered logistic regression model predicting intersubjective social status based on information written in the profile and characteristics of the respondents. Finally, Model 4 refers to the model that adds the interaction terms of the information written in the profile and the characteristics of the respondent to Model 3.

**Table 6:** Multi-level ordered logistic regression models predicting intersubjective social status.

	Model 1	Model 2	Model 3	Model 4
Profile				
Women	0.291∗∗∗	0.405∗∗∗	0.408∗∗∗	0.405∗∗∗
	(0.069)	(0.085)	(0.085)	(0.085)
Married	0.673∗∗∗	1.010∗∗∗	1.010∗∗∗	1.004∗∗∗
	(0.069)	(0.086)	(0.086)	(0.086)
College Graduates	0.760∗∗∗	1.152∗∗∗	1.159∗∗∗	1.187∗∗∗
	(0.069)	(0.088)	(0.088)	(0.122)
Regular Employment	1.437∗∗∗	2.301∗∗∗	2.301∗∗∗	2.179∗∗∗
	(0.073)	(0.098)	(0.098)	(0.121)
Respondents				
Women			0.372∗	0.373∗
			(0.189)	(0.189)
College Graduates			−0.603∗∗∗	−0.576∗∗
			(0.175)	(0.198)
Regular Employment			−0.510∗∗	−0.667∗∗∗
			(0.175)	(0.198)
College(P) × College(R)				−0.054
				(0.168)
Regular(P) × Regular(R)				0.285
				(0.168)
SD (Intercept)		2.214	2.158	2.160

1|2	−1.963∗∗∗	−3.269∗∗∗	−3.662∗∗∗	−3.727∗∗∗
	(0.113)	(0.175)	(0.224)	(0.234)
2|3	0.433∗∗∗	0.593∗∗∗	0.193	0.137
	(0.081)	(0.129)	(0.188)	(0.198)
3|4	2.803∗∗∗	4.470∗∗∗	4.072∗∗∗	4.018∗∗∗
	(0.096)	(0.163)	(0.209)	(0.217)
4|5	5.927∗∗∗	9.066∗∗∗	8.666∗∗∗	8.611∗∗∗
	(0.161)	(0.266)	(0.292)	(0.299)
Num. Obs.	3205	3205	3205	3205
Num. Groups.	804	804	804	804
AIC	7311.4	6377.5	6351.4	6352.4
BIC	7360.0	6432.2	6424.3	6437.5
RMSE	2.93	2.56	2.56	2.56

+ p < 0.1, ∗p < 0.05, ∗∗p < 0.01, ∗∗∗p < 0.001.

The Akaike information criterion (AIC) and Bayesian information criterion (BIC) show the lowest values in Model 3. Considering the AIC and BIC, Model 3 can be adopted as the model that best fits the data from the online survey experiment among the four models. In Model 3, gender (women vs. men), marital status (married vs. unmarried), education level (college graduate or high school), and employment status (regular or non-regular employment) at the profile level were significantly associated with intersubjective social status. Furthermore, the variables of gender (women or men), educational level (college graduate or not), and employment status (regular employment or not) at the respondent level were significantly associated with intersubjective social status.

Three cutoff points (1|2, 3|4, and 4|5) were statistically significant, but one cutoff point (2|3) was not. This implies that intersubjective social status is hierarchically ordered, whereas the difference between two (upper-low) and three (lower-middle) is relatively vague. Table 6 provides further evidence that the coefficient of regular employment at the profile level has the highest value in Model 3. This means that employment status has the most significant impact on intersubjective social status compared to other variables (gender, marital status, and education). Finally, advantaged individuals (men, highly educated, and regularly employed) were less likely to positively rate the social status of individuals in the presented profile. To address selection bias in the experimental data, which overrepresented women and unemployed individuals, I created weighting variables based on SSP2022 and re-estimated the models using these variables. Consequently, the analytical results of models adjusted based on weight variable are largely consistent with the original results (see Table S2 in Supplemental Materials in detail). This finding is consistent with those of previous studies(Chen & Fan, 2015; Davis & Robinson, 1988; Knell & Stix, 2020; Sudo, 2021).

The findings based on the ordered logistic regression model and multilevel ordered logistic regression model show that the structures of subjective and intersubjective social statuses share basic features. For instance, marital status has statistically significant meanings for both. Regarding objective social status, educational level and employment status were commonly associated with subjective and intersubjective social statuses. This implies that individuals tend to construct subjective and intersubjective social statuses using similar methods.

However, the structures of subjective and intersubjective social statuses do not coincide perfectly. Educational level as well as employment status are significantly associated with subjective social status, and their associations do not differ significantly. For intersubjective social status, employment status is more significantly associated with intersubjective social status, compared to education level. Here, it is assumed that education is mainly related to the social inequality of opportunities, whereas employment status is mainly related to the social inequality of outcomes(Hwang, 2024). Although subjective social status is related to the social inequality in opportunities, intersubjective social status is primarily related to the social inequality in outcomes.

Additionally, the results of the multilevel ordered logistic regression model revealed that the respondent's characteristics tended to bias intersubjective social status. Specifically, advantaged individuals (men, highly educated, or regularly employed) are less likely to positively rate others' social classes (intersubjective social status). If this tendency is also observed in self-rated social class (subjective social status), advantaged people are likely to attribute themselves to relatively lower-status groups. Although this finding does not seem obvious, it is consistent with the findings of previous studies(Chen & Fan, 2015; Davis & Robinson, 1988; Kim et al., 2018; Knell & Stix, 2020; Sudo, 2021). Future studies should carefully explore why advantaged individuals do not tend to rate others' social status positively.

## 6 Discussion and conclusions

### 6.1 Discussion

In this study, I inferred that subjective and intersubjective social statuses share basic features (Hypothesis 1). The analytical results revealed that marital status, educational level, and employment status were significantly and commonly associated with subjective and intersubjective social statuses. This finding supports Hypothesis 1, and it can be concluded that subjective and intersubjective social statuses are constructed based on objective social status in similar ways.

Moreover, I inferred that socially advantaged individuals are more likely to lower their subjective social status (Hypothesis 2). The analytical results suggest that socially advantaged individuals (men, highly educated, and regularly employed) are more likely to rate their intersubjective social status lower than socially disadvantaged individuals (women, not highly educated, and those in irregular employment). The analytical results will support Hypothesis 2, and it can be concluded that the social implications of objective social status might differ depending on differences between social status groups.

In other words, demographic and socioeconomic characteristics are related to the discrepancies between subjective and objective social statuses (or intersubjective social status). Individuals belonging to high-status groups tend to underestimate their status, whereas individuals belonging to disadvantaged status groups tend to overestimate their status (Park et al., 2024). Thus, the social implication of high status is not the same across social-status groups. Consequently, this results in the problems faced by disadvantaged people—stemming from objective social inequalities—being overlooked, because advantaged individuals tend to underestimate the extent of inequality in their society.

Finally, I inferred that subjective social status is more strongly related to the social inequality of opportunities, compared to intersubjective social status (Hypothesis 3). The analytical results showed that subjective social status was associated with both educational level and employment status, but intersubjective social status was mainly associated with employment status. The analytical results support Hypothesis 3, and it is concluded that subjective and intersubjective social statuses might be differently associated with social inequalities.

Although intersubjective social status is mainly related to the social inequality of outcomes, subjective social status is more strongly related to the social inequality of opportunities (Hwang, 2024). This difference likely reflects the types of information used to determine intersubjective and subjective social statuses. For others, information on the current status (outcomes) of individuals is easily observable, but information on their past status (opportunities) is difficult to observe. However, for the individuals themselves, the past status of themselves remains relatively observable. Others tend to judge an individual's social status based only on current circumstances; in contrast, individuals themselves assess their status using past, current, and (potential) future statuses. Moreover, as subjective social status is only weakly associated with (current) objective social status, it is concluded that social inequality observed in subjective social status is only weakly associated with social inequality observed in (current) objective social status (Schmalor & Heine, 2022).

In the literature, it was clarified that subjective social status is associated with social inequalities. However, previous studies could not specify what type of social inequality subjective social status emphasizes. This study clarifies that compared to intersubjective social status, subjective social status emphasizes not only social inequality of outcomes but also social inequality of opportunities. This implies that distinguishing between subjective social status and intersubjective social status is highly significant when investigating social inequalities. Specifically, by using the concept of intersubjective social status effectively, social researchers can distinguish between social inequality of outcomes (measured by intersubjective social status) and social inequality of opportunities (measured by subjective social status and intersubjective social status). Hence, social researchers and social policymakers are positioned to address the measurement and reduction of social inequalities of opportunity. For instance, it is known that subjective social status is more strongly associated with subjective health than objective social status. This implies that reducing inequality of (educational) opportunity might be more effective than the redistribution of wealth for improving average subjective well-being in society.

### 6.2 Limitations

First, subjective (or intersubjective) social status was measured by only a single inquiry term on a 5-point Likert scale. However, previous studies have stated that subjective social status cannot be estimated one-dimensionally because it is constructed multidimensionally depending on various social contexts (Kluegel et al., 1977; Raudenská, 2024). For instance, subjective social status may be measured based not only on individual status but also on family status(Davis & Robinson, 1988, 1998; Luo & Brayfield, 1996; Yamaguchi & Wang, 2002). The social context determines which unit (individual or family) is more suited to estimate subjective social status (Dimova & Dimov, 2023; Kim & Lee, 2021). To capture the multidimensional characteristics of subjective social status, subjective social status has been estimated by utilizing multi-dimensional measurement scale or domain-specific measure (e.g., occupational prestige, household income, and neighborhood relation) in future.

Second, the experimental conditions used to measure intersubjective social status were limited. In the online survey experiment, I established four conditions: male versus female, married versus unmarried, college graduate versus high school graduate, and regular employment versus non--regular employment. The age of the participants in the fictional profiles was fixed at 35 years. Similarly, the jobs of the individuals written in the fictional profiles were fixed as those of office workers. However, these conditions may be insufficient to accurately measure intersubjective social status. As an avenue for future research, the robustness of this study's findings under different experimental conditions and age cohorts should be tested.

Third, selection bias should be carefully considered in the data from the online survey experiment used in this study. As I adopted an online survey method, the respondents in the dataset consisted of online monitors registered with a research agency in Japan. In other words, because I did not follow a random sampling method, the sample of the online survey experiment could potentially include selection biases. Additionally, differences between cultural contexts (or social regimes) must be considered. For instance, the regular/non-regular employment distinction in Japan is highly specific. In the social context of Japan, non-regular employment status is significantly more fragile compared to regular employment status, and upward mobility to regular employment often proves difficult for non-regular employees. It is possible that the findings in Japanese society (obtained through online monitors) might not be directly applicable to other societies. To overcome this limitation, the experiment should be replicated based on random sampling data across countries.

Despite these limitations, the study succeeded in revealing how and why subjective social status is associated with social inequalities. This insight contributes to a deeper understanding of strategies for reducing perceived social inequality, which is distinct from objective social inequality.

### 6.3 Conclusions

This study proposed the concept of intersubjective social status. Accordingly, I clarified that intersubjective social status may vary depending on social-status groups. This suggests that the theory of the reference group may explain the discrepancy between subjective and objective social statuses. Notably, differences in subjective and intersubjective social statuses are related to social inequality. The analytical results imply that subjective social status is associated more strongly with the social inequality of opportunities than with the social inequality of outcomes, and vice versa for intersubjective social status. Previous studies (Bavetta et al., 2019; Trump, 2023) have clarified that objective and subjective social inequality may not coincide. That discrepancy between subjective social inequality and objective social inequality could be explained by considering differences in subjective and intersubjective (or objective) social statuses.

## Ethical statement

The online survey experiment conducted in this study was approved by Hitotsubashi University Institutional Review Board (Approval Number: 2024C054).

## Code availability

Contact the corresponding author. The author will open the code on GitHub.

## Ethical statement

This manuscript has not been published or presented elsewhere in part or in entirety and is not under consideration by another journal. The study design was approved by the appropriate ethics review board. I have read and understood your journal's policies, and I believe that neither the manuscript nor the study violates any of these. There are no conflicts of interest to declare.

## Funding

This research is supported by the Grants-in-Aid for Scientific Research of Japan Society for the Promotion of Science (19H00609, 21H00776, 23K25637, 24K05251) and HIAS Brain Research Center (https://brc.hias.hit-u.ac.jp/en/).

## Declaration of competing interest

The authors declare that they have no known competing financial interests or personal relationships that could have appeared to influence the work reported in this paper.

## Data Availability

The experimental data is available upon request to the corresponding author. The author will open the data on GitHub. The SSP2022 data is available upon request to the SSP Project (https://ssp.hus.osaka-u.ac.jp/). The SSP Project allowed me to use the SSP2022 data.

## APPENDIX . Fictional Profile used in the Online Experiment (example)

Question. When Japanese society is categorized into five strata, could you tell me how you expect Taro Yamada to judge his social stratum?

Name: Taro Yamada.

[Profile]

**Age**: 35 years.

**Education**: **College graduate**.

**Family**: **Unmarried**.

**Occupation**: Office clerk (product planning and development)

**Employment status**: **Regular**.

Answer.

● Upper
● Upper-Middle
● Lower-Middle
● Upper-Lower
● Lower-Lower
