# Supplementary material for: Subjective, intersubjective, and objective social statuses: How do people imagine social inequality?
Source: SSM Popul Health. 2025 Nov 8;32:101878. doi: 10.1016/j.ssmph.2025.101878 (PMC12664347; doi:10.1016/j.ssmph.2025.101878)
Supplement: Multimedia component 1 [file mmc1.docx]

SUPPLEMENTAL MATERIALS

**Table A1**. Results of Ordered Logistic Regression Model Predicting Subjective Social Status (full sample)

|  | Model 1 |
| --- | --- |
| Women | 0.132 |
|  | (0.085) |
| Nonbinary | 1.103+ |
|  | (0.670) |
| Married | 0.991*** |
|  | (0.094) |
| Divorce/Bereaved | 0.154 |
|  | (0.162) |
| College Graduate | 1.031*** |
|  | (0.081) |
| Regular Employment | 0.543*** |
|  | (0.102) |
| Self-employed | 0.268 |
|  | (0.165) |
| No Job | 0.272* |
|  | (0.138) |
| No Information for Employment | 0.434* |
|  | (0.218) |
| 1\|2 | -1.367*** |
|  | (0.143) |
| 2\|3 | 0.323* |
|  | (0.129) |
| 3\|4 | 2.475*** |
|  | (0.140) |
| 4\|5 | 5.834*** |
|  | (0.204) |
| Num. Obs. | 2387 |
| AIC | 5725.4 |
| BIC | 5800.5 |
| RMSE | 3.00 |
| + p < 0.1, * p < 0.05, ** p < 0.01, *** p < 0.001 | |

**Table S2**. Multi-Level Ordered Logistic Regression Models Predicting Intersubjective Social Status (weighted)

|  | Model 1 | Model 2 | Model 3 | Model 4 |
| --- | --- | --- | --- | --- |
| Profile |  |  |  |  |
| Women | 0.323*** | 0.460*** | 0.464*** | 0.462*** |
|  | (0.069) | (0.086) | (0.085) | (0.085) |
| Married | 0.668*** | 0.931*** | 0.932*** | 0.928*** |
|  | (0.069) | (0.086) | (0.086) | (0.086) |
| College Graduates | 0.844*** | 1.283*** | 1.291*** | 1.254*** |
|  | (0.070) | (0.089) | (0.089) | (0.117) |
| Regular Employment | 1.485*** | 2.391*** | 2.391*** | 2.296*** |
|  | (0.073) | (0.099) | (0.099) | (0.128) |
| Respondents |  |  |  |  |
| Women |  |  | 0.361+ | 0.361+ |
|  |  |  | (0.186) | (0.186) |
| College Graduates |  |  | -0.663*** | -0.709*** |
|  |  |  | (0.178) | (0.201) |
| Regular Employment |  |  | -0.562** | -0.673*** |
|  |  |  | (0.179) | (0.202) |
| College(P) × College(R) |  |  |  | 0.083 |
|  |  |  |  | (0.170) |
| Regular(P) × Regular(R) |  |  |  | 0.197 |
|  |  |  |  | (0.168) |
| SD (Intercept) |  | 2.119 | 2.045 | 2.045 |
| 1\|2 | -2.191*** | -3.311*** | -3.749*** | -3.834*** |
|  | (0.125) | (0.181) | (0.237) | (0.247) |
| 2\|3 | 0.431*** | 0.660*** | 0.214 | 0.138 |
|  | (0.081) | (0.131) | (0.197) | (0.207) |
| 3\|4 | 2.869*** | 4.591*** | 4.146*** | 4.071*** |
|  | (0.098) | (0.166) | (0.218) | (0.226) |
| 4\|5 | 6.085*** | 9.355*** | 8.907*** | 8.825*** |
|  | (0.164) | (0.274) | (0.305) | (0.312) |
| Num. Obs. | 3205 | 3205 | 3205 | 3205 |
| Num. Groups. | 804 | 804 | 804 | 804 |
| AIC | 7109.6 | 6114.0 | 6081.4 | 6083.7 |
| BIC | 7158.1 | 6168.6 | 6154.2 | 6168.7 |
| RMSE | 2.93 | 2.58 | 2.58 | 2.58 |
| + p < 0.1, * p < 0.05, ** p < 0.01, *** p < 0.001 | | |  |  |
